# Cutaneous Respirometry as Novel Technique to Monitor Mitochondrial Function: A Feasibility Study in Healthy Volunteers

**DOI:** 10.1371/journal.pone.0159544

**Published:** 2016-07-25

**Authors:** Floor Harms, Robert Jan Stolker, Egbert Mik

**Affiliations:** 1 Department of Anesthesiology, Laboratory of Experimental Anesthesiology, Erasmus University Medical Center Rotterdam, ‘s-Gravendijkwal 230, 3015 CE Rotterdam, The Netherlands; 2 Department of Intensive Care, Erasmus University Medical Center Rotterdam, ‘s-Gravendijkwal 230, 3015 CE Rotterdam, The Netherlands; University of Pecs Medical School, HUNGARY

## Abstract

**Background:**

The protoporphyrin IX-triplet state lifetime technique (PpIX-TSLT) is proposed as a potential clinical non-invasive tool to monitor mitochondrial function. This technique has been evaluated in several animal studies. Mitochondrial respirometry allows measurement *in vivo* of mitochondrial oxygen tension (mitoPO_2_) and mitochondrial oxygen consumption (mitoVO_2_) in skin. This study describes the first use of a clinical prototype in skin of humans.

**Methods:**

The clinical prototype was tested in 30 healthy volunteers. A self-adhesive patch containing 2 mg 5-aminolevulinic acid (ALA) was applied on the skin of the anterior chest wall (sternal) for induction of mitochondrial protoporphyrin IX and was protected from light for 5 h. MitoPO_2_ was measured by means of oxygen-dependent delayed fluorescence of protoporphyrin IX. MitoVO_2_ was determined by dynamic mitoPO_2_ measurements on the primed skin, while locally blocking oxygen supply by applying local pressure with the measurement probe. MitoPO_2_ was recorded before and during a 60-s period of compression of the microcirculation, at an interval of 1 Hz. Oxygen consumption (i.e. the local oxygen disappearance rate) was calculated from the decay of the mitoPO_2_ slope.

**Results:**

Oxygen-dependent delayed fluorescence measurements were successfully performed in the skin of 27 volunteers. The average value (± SD) of mitoPO_2_ was 44 ± 17 mmHg and mean mitoVO_2_ values were 5.8 ± 2.3 and 6.1 ± 1.6 mmHg s^-1^ at a skin temperature of 34°C and 40°C, respectively. No major discomfort during measurement and no long-term dermatological abnormalities were reported in a survey performed 1 month after measurements.

**Conclusion:**

These results show that the clinical prototype allows measurement of mitochondrial oxygenation and oxygen consumption in humans. The development of this clinically applicable device offers opportunities for further evaluation of the technique in humans and the start of first clinical studies.

## Introduction

An adequate supply of oxygen to tissue and its subsequent use in oxidative phosphorylation in the mitochondria is essential for preserving cellular integrity and, ultimately, life. Therefore, a non-invasive and *in vivo* monitoring system able to monitor mitochondrial parameters, like oxygenation and oxygen consumption, could be a valuable tool for clinicians [1].

The most common *ex vivo* techniques are oxygen consumption measurements using oxygen electrodes [2], such as high-resolution respirometry [3]. These *ex vivo* techniques measure in suspensions of isolated cells and mitochondria, or small tissue biopsies and may, therefore, not adequately reflect the *in vivo* situation. Another method, the XF24 Extracellular Flux Analyze (Seahorse Bioscience) [4, 5] allows *ex vivo* measuring of mitochondrial oxygen consumption in intact cells or tissue by oxygen-sensing fluorophores. Although these approaches provide highly specific information on the function of the mitochondrial respiratory chain, the *ex vivo* use is a well-recognized limitation [6]. *Ex vivo* techniques are incapable for bed-side montoring and determination of mitochondrial function under physiological or pathophysiological circumstances is essential to understand and evaluate changes in oxidative phosphorylation [7].

To overcome limitations of *ex vivo* approaches, numerous *in vivo* techniques have been used to study mitochondrial function within the context of the physiological environment. Most commonly used methods to measure mitochondrial function *in vivo* are Nuclear Magnetic Resonance (NMR) spectroscopy and near-infrared spectroscopy (NIRS). NMR spectroscopy measures changes in phosphormetabolites to determine rates of resting and maximal mitochondrial ATP production. Unfortunately, the need of a Magnetic Resonance Imaging (MRI) scan makes NMR spectroscopy expensive and unable for bed-side monitoring. In contrast, NIRS enables bedside monitoring of mitochondrial function by continuous monitoring of variations in hemoglobin oxygenation and in the redox state of cytochrome c oxidase. A new application of NIRS allows measurement of skeletal muscle oxidative capacity by following the change in the rate of oxygen consumption during recovery from ischemia or exercise [8–10]. Unfortunately, recovery of the cytochrome oxidase signal from NIRS data remains controversial [11]. NIRS is influenced by tissue-specific effects, such as the wavelength dependence of the optical path length and changes in light scattering [12, 13].

Indirect calorimetry is the current golden standard for examining respiration *in vivo*. This technique measures inspired and expired gas flows, volumes and concentrations of oxygen (O_2_) and carbon dioxide (CO_2_), and allows measurement of total body oxygen consumption (VO_2_). Also Fick’s principle has been applied in humans for many years to measure VO_2_ by combining data of regional blood flow and arterial-venous oxygen content difference. However, both measurements can generate data with a high variability and, therefore, its use for clinical decision-making has been questioned [14, 15].

The protoporphyrin IX-triplet state lifetime technique (PpIX-TSLT) is proposed as a potential novel approach to determine mitochondrial oxygen consumption [16]. The first detailed description of the PpIX-TSLT was published in 2006 [17]. PpIX-TSLT enables mitochondrial oxygen (mitoPO_2_) measurements by means of the oxygen-dependent optical properties of protoporphyrin IX (PpIX). PpIX-TSLT was the first technique to allow measurements of mitoPO_2_ in living cells and can be applied *in vivo* [18–20]. Our group has been working on the development of PpIX-TSLT from its use in cell cultures to a monitoring system of mitochondrial function in humans [21]. This technique has been tested and calibrated for use on isolated organs [19, 20] and *in vivo* [20]. Subsequently, oxygen-dependent quenching of delayed fluorescence of PpIX has been observed in skin [18] and validation of the quenching constants needed to calculate mitoPO_2_ from the signals has been successful [22]. In addition to direct non-invasive measurement of mitoPO_2_, it is also technically possible to gain information on mitochondrial function. Information on mitochondrial oxygen consumption (mitoVO_2_) can be obtained by dynamic mitoPO_2_ measurements while blocking local oxygen supply. The local oxygen disappearance rate is a measure of oxygen consumption and can be calculated from the decay of the mitoPO_2_ [23]. As proposed earlier [18, 22–26], it should be technically possible and safe to apply PpIX-TSLT in humans.

In the present study, we demonstrate for the first time the ability to measure mitoPO_2_ and mitoVO_2_ in human skin, using a clinical prototype of PpIX-TSLT. The safety and feasibility of our method is investigated and data are presented on the inter- and intra-individual distribution of mitochondrial oxygen measurements. Our ultimate goal is to develop the PpIX-TSLT as a non-invasive monitor that allows direct assessment of mitochondrial oxygenation and (dys)function in critically ill patients. Although mitochondrial dysfunctions are thought to be related to the pathogenesis of sepsis and multiorgan failure [26–28], inconsistent data have been reported. These conflicting results may be due to the wide variety of methods used to determine mitochondrial dysfunction in sepsis [1]. Therefore, a standard method to monitor mitochondrial dysfunction could further elucidate the role of the mitochondria during septic conditions. When the PpIX-TSLT has proven to do this successfully, opportunities will arise for novel strategies in the treatment of severe sepsis.

## Methods

The study was performed in compliance with the Code of Ethics of the World Medical Association (Declaration of Helsinki) and approved by the Institutional Review Board at the Erasmus MC CCMO-register NL37911.078.11). A total of 30 volunteers were drawn from a pool of young, healthy students and hospital staff aged 18–30 years. Written informed consent was obtained from all volunteers prior to study participation.

### Principle of mitoPO_2_ measurements

The background of the PpIX-TSLT is described in detail elsewhere [17, 20]. In short, PpIX is the final precursor of heme in the heme biosynthetic pathway. PpIX is synthesized in the mitochondria, and administration of 5-aminolevulinic acid (ALA) substantially enhances the PpIX concentration. PpIX possesses a triplet state that reacts strongly with oxygen, making its lifetime oxygen-dependent. Population of the first excited triplet state occurs upon photo-excitation with a pulse of light, and causes the emission of red delayed fluorescence. The delayed fluorescence lifetime is related to mitoPO_2_ according to the Stern-Volmer equation:

$$mitoPO_{2}=\left(1/\tau -1/\tau_{0}\right)/k_{q}$$ (1)

in which *τ* is the measured delayed fluorescence lifetime, *k*_*q*_ is the quenching constant and *τ*_*0*_ is the lifetime at zero oxygen. Eq 1 is valid for a homogenous oxygen distribution and after excitation with a pulse of light of which the lifetime is much shorter than *τ* [29]. In case of a non-homogenous oxygen distribution inside the measurement volume, a reliable estimation of the average PO_2_ can be made by the rectangular distribution method (RDM) [30, 31].

The signal/noise ratio (SNR) of resulting traces was calculated and defined as the ratio of maximum signal amplitude to the peak-to-peak noise. Lifetime analysis operates stably at moderate SNR (SNR >20) [20]. Therefore, only delayed fluorescence signals with a SNR >20 were analyzed and included in the present dataset.

### Principle of mitoVO_2_ measurements

**Fig 1:**
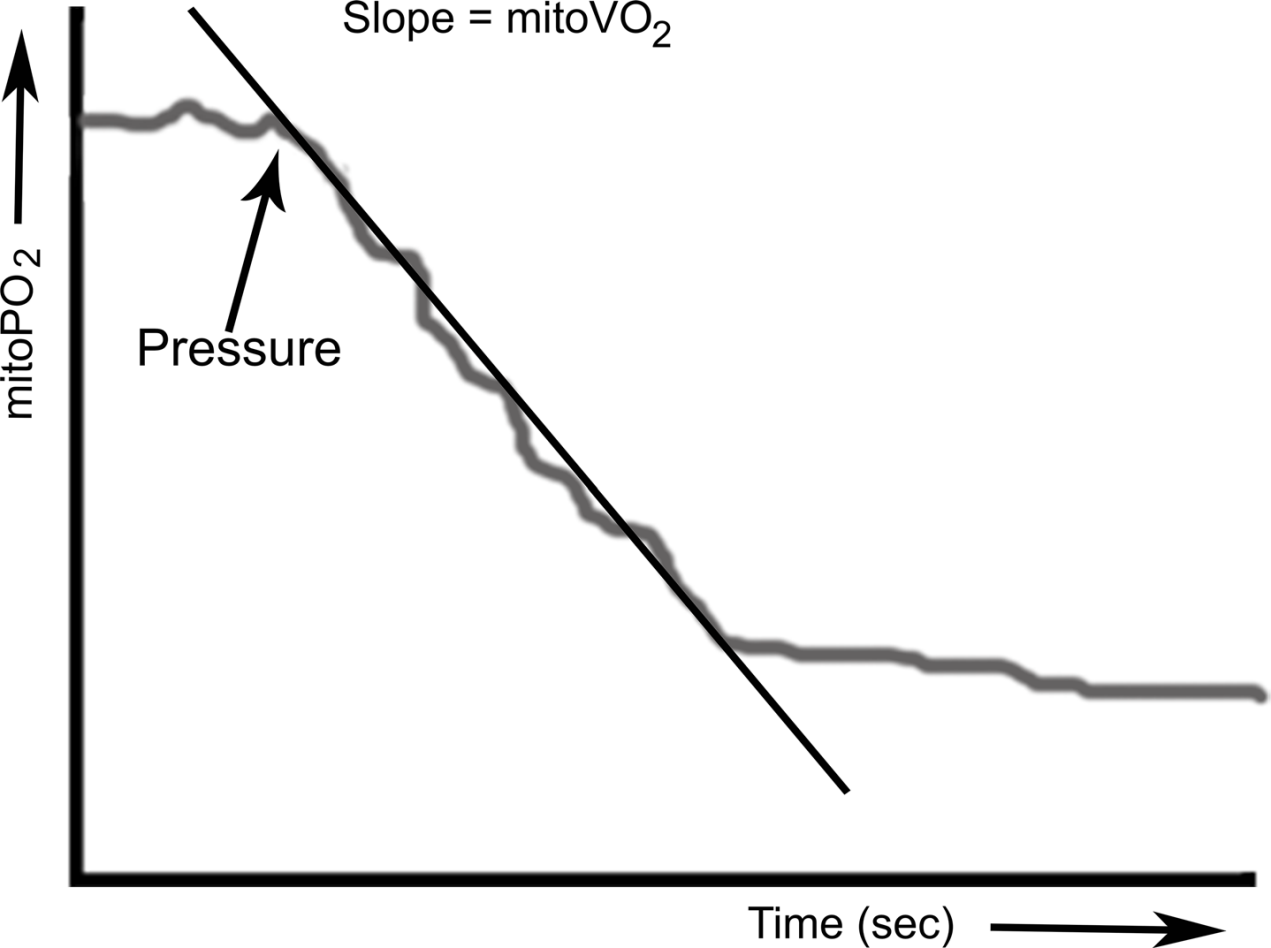
Principle of respirometry by local cessation of the oxygen supply. Mitochondrial oxygen consumption (mitoVO_2_) is calculated from the linear part of the oxygen disappearance curve following local compression of the microcirculation by the measurement probe.

MitoVO_2_ is measured by means of the oxygen disappearance rate (ODR) after local occlusion of the oxygen supply. Local occlusion of the microcirculation in skin was obtained by application of pressure with the measurement probe. This simple procedure created reliable stop-flow conditions and induced measurable oxygen disappearance rates, due to cessation of microvascular oxygen supply and ongoing cellular oxygen consumption. MitoPO_2_ was measured before and during application of pressure at an interval of 1 Hz, using 4 laser pulses per measurement. We have described the basic principles behind the technology and have provided a working implementation of the technique for mitoVO_2_ measurements (15) as well as a method to calculate mitoVO_2_. Fig 1 presents an example of the analysis of mitoVO_2_.

### Experimental setup

Prior to oxygen measurements the heart rate, blood pressure and peripheral oxygen saturation were determined in all volunteers, and a standard preoperative evaluation form was obtained from all these participants.

Oxygen measurements were performed by means of a clinical prototype PpIX-TSLT device [24]. A self-adhesive patch containing 8 mg ALA (Alacare^®^; Spirig AG, Egerkingen, Switzerland) was applied on the skin of the anterior chest wall (sternal), for induction of PpIX. To enhance ALA penetration adequate skin preparation proved essential. Hair was shaved (if present) and the skin was rubbed with a fine abrasive pad of a standard ECG sticker to remove the top parts of the stratum corneum. After ALA application, the skin was protected from light for 5 h. During these 5 h a suitable concentration of PpIX was synthesized to enable measurements of mitoPO_2_ and mitoVO_2_. To minimize the influence from ambient light, the experiments were performed in dimmed light. First, three successive mitoPO_2_ measurements were performed during a period of 90 s under baseline conditions. Subsequently, mitoVO_2_ was determined with a heated measurement probe at 34°C and 40°C. Warming of the measurement probe was done to prevent local vasoconstriction due to a cold probe, and to eliminate the effect of differences in skin temperature on the measurements_._ The temperature of 34°C was chosen because this was the maximal skin temperature determined in 10 healthy volunteers in a normal environment, measured by infrared thermography. To determine the effect of hyperthermia on mitoVO_2_ a probe temperature of 40°C was chosen. This temperature was well above the mean skin temperature [32] and low enough not to induce skin burns. One month after the experiments an evaluation form was completed by all volunteers. The form consisted of questions about any pain during the measurements and the appearance of dermatological symptoms, such as erythema, pruritus and hyperpigmentation of the skin.

### Statistical analysis

Unless stated otherwise, reported values are mean ± SD. Normality of the data was tested using the Shapiro-Wilk test. Correlation between the two variables was tested by Pearson's correlation analysis.

## Results

Basic characteristics of the 30 healthy volunteers are shown in Table 1. All participants had normal results for the physical parameters, such as heart rate, blood pressure and arterial oxygen saturation. Due to insufficient signal-to-noise ratio the measurements of three volunteers were excluded from further analysis.

**Table 1:** Characteristics healthy volunteers.

Characteristics		Volunteers (n = 30)
Age (years)		26 ± 5
Sex, n (%)		
	Men	19 (63)
	Women	11 (37)
Heart rate (bpm ± SD)		69 ± 13
Systolic blood pressure (mmHg ± SD)		128 ± 12
Diastolic blood pressure (mmHg ± SD)		76 ± 10
Arterial saturation (% ± SD)		99 ± 1

### MitoPO_2_ Measurements

**Fig 2:**
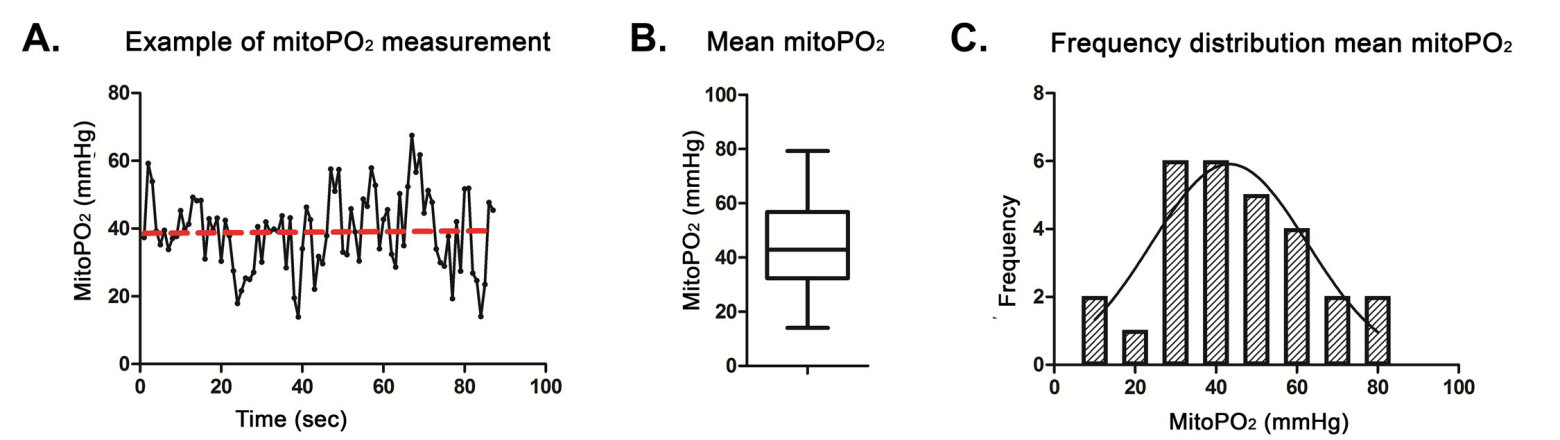
**A.** Example of a baseline mitoPO_2_ measurement. The mean mitochondrial oxygen tension (mitoPO_2_) (red line) is calculated over a period of 90 second. **B.** The mitoPO_2_ is presented in a box-and-whisker graph, **C.** Frequency distribution of all ratio differences of the mitoPO_2._

A typical example of a baseline mitoPO_2_ measurement is presented in Fig 2A. Due to the heterogeneous oxygenation in the skin a marked intra-individual variation was observed during the baseline measurement due to movement (breathing) in combination with a small measuring area (the diameter of the detection fiber was only 1 mm). Therefore, the mean mitoPO_2_ (red line) over a period of 90 s was used in the analysis of mitoPO_2._

From the total of 27 experiments, mean mitoPO_2_ was 44 ± 17 mmHg (Fig 2B). This finding is consistent with previous values derived from animal studies [21, 33]. Despite the movement-induced variation in baseline mitoPO_2_, the mean data regress towards a normal distribution (p = 0.73; skewness = 0.07 ± 0.45, kurtosis = -0.46 ± 0.87) (Fig 2C).

### MitoVO_2_ Measurements

**Fig 3:**
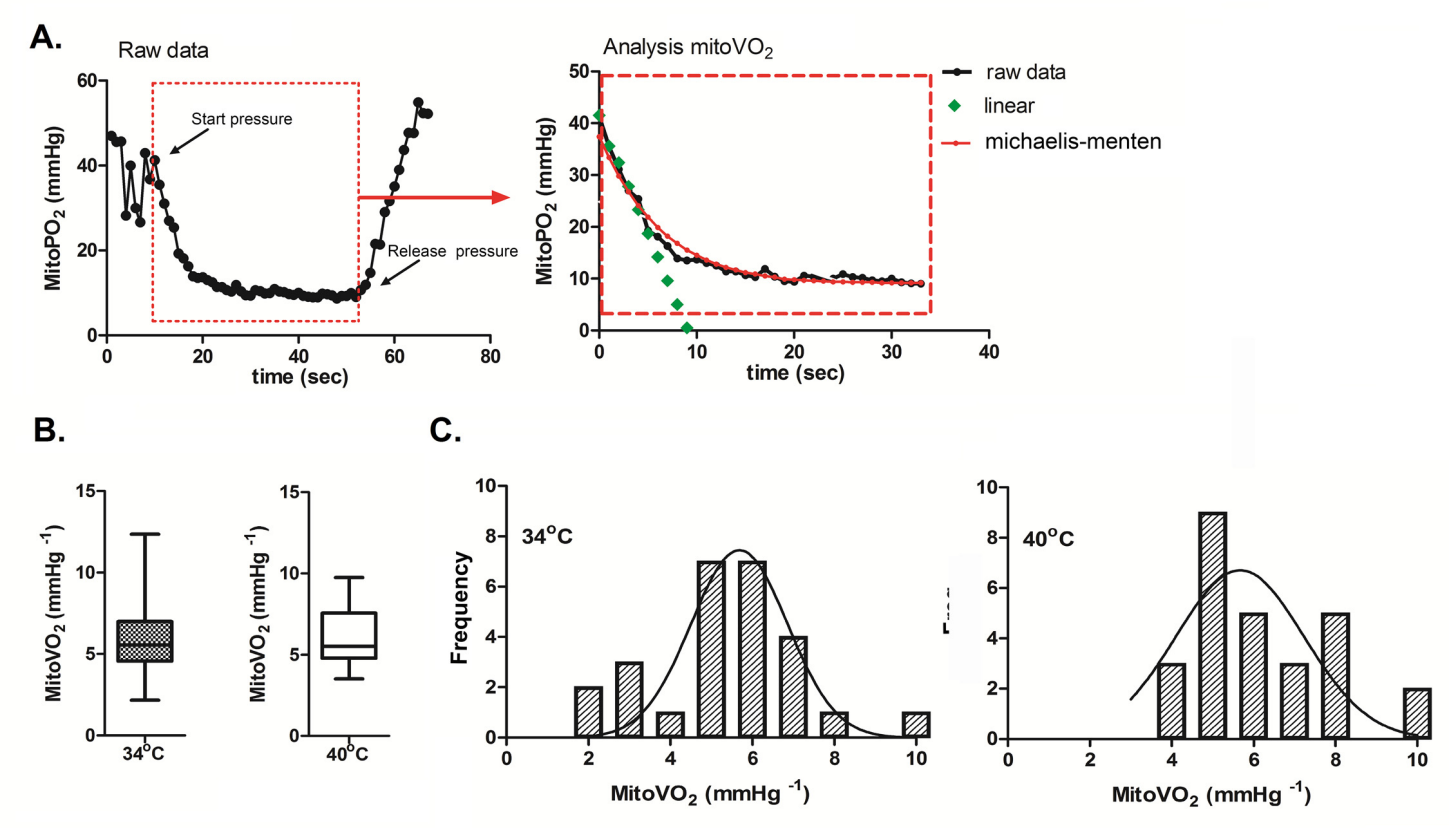
**A**. Typical example of the analysis of the mitochondrial oxygen consumption (mitoVO_2_). The first panel shows an example of the oxygen disappearance rate after local occlusion of the microcirculation. In the second panel we demonstrate the analysis of the mitochondrial oxygen tension (mitoPO_2_), the green line represents the linear fit (ΔPO_2_(t)/Δt) of the oxygen disappearance rate. **B**. The mitoVO_2_ are presented in a box-and-whiskers graph. The boxes extend from the 25^th^ percentile to the 75^th^ percentile, with a line at the median, the whiskers extend above and below the box to show the highest and lowest values. Presented data are measured with two different probe temperatures (34°C and 40°C, respectively). **C.** Frequency distribution of all ratio differences of the mitoVO_2_ at 34°C and 40°C.

A typical example of an oxygen disappearance curve on human skin is presented in Fig 3A. The mean mitoVO_2_ values, measured in the skin of 27 healthy volunteers, were 5.8 ± 2.3 and 6.1 ± 1.6 mmHg s^-1^ for a probe temperature of 34°C and 40°C, respectively (Fig 3B). The distribution of the data is presented in Fig 3C. The Shapiro-Wilk test demonstrates that mitoVO_2_ measured at a temperature of 34°C follows a normal distribution (p-value = 0.069, skewness = 0.9, kurtosis = 1.9). However, the same analysis of mitoVO_2_ at a temperature of 40°C does not follow a normal distribution (p-value = 0.047, skewness = 0.72 ± 0.45, kurtosis = -0.29 ± 0.87).

### Correlation between mitoPO_2_ and mitoVO_2_

**Fig 4:**
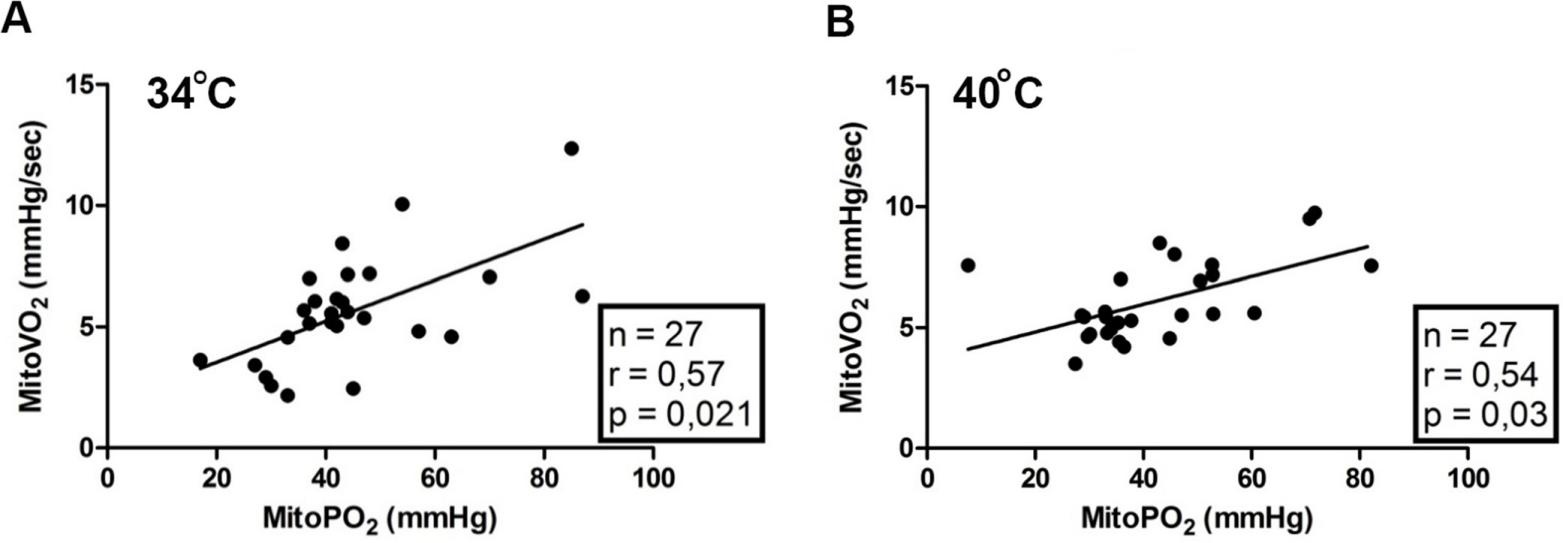
**Correlation plot of the initial mitochondrial oxygen tension (mitoPO_2_) and the mitochondrial oxygen consumption (mitoVO_2_) at 34°C (A) and 40°C (B)**.

We found a weak positive correlation between mitoVO_2_ and baseline mitoPO_2_ for 27 data pairs (Fig 4). This indicates that mitoVO_2_ of the skin might not be completely independent of baseline mitoPO_2_ levels. However, the correlation is mainly caused by very high and/or very low mitoPO_2_ values.

### Safety of the measurements

All healthy volunteers experienced the skin preparation and measurements as non-problematic. Due to either the skin preparation or the ALA patch, 45% of the volunteers suffered from mild pruritus and/or erythema on the actual measurement day; these minor complaints were no longer present the day after the measurements. Only two volunteers had transient hyperpigmentation of the skin after the measurements, possibly due to premature exposure of the primed area to sunlight (against our advice). The hyperpigmentation was temporary and disappeared within one month. None of the volunteers sustained long-term skin damage, as established one month after the experiments.

## Discussion

This study presents the first results of a clinical prototype of a novel non-invasive monitoring device based on PpIX-TSLT. This device enables measurement of mitochondrial oxygenation and oxygen consumption in human skin. The PpIX-TSLT appears to be a feasible and safe non-invasive measurement tool that allows performing a functional optical biopsy in intact skin.

Following topical application of ALA on the skin above the sternum, after 5 h we could detect delayed fluorescence signals, of which the lifetime kinetics could be analyzed in 27 of the 30 healthy volunteers. Three volunteers were excluded from the analysis because the SNR was insufficient; this was caused by technical problems with the light source at the time of measurement (laser output unstable and with reduced intensity). Mean mitoPO_2_ values were around 45 mmHg. This is slightly lower than previously measured in rats [24, 26] and is probably explained by inter-species differences, as well as differences in the fractional inspired oxygen concentration (FiO_2_). The FiO_2_ in rats was 0.40 versus an atmospheric FiO_2_ in healthy volunteers.

An intra-person variation in mitoPO_2_ was observed during the mitoPO_2_ measurements. Small movements of the measuring probe relative to the skin can lead to changes in mitoPO_2_ due to the small measurement volume in a heterogenic tissue surface. Therefore, our group is currently working on a measuring probe with revised optics to increase the detection area on the skin. Combined with the ability to be placed flat on the skin and be affixed above the measuring spot, this improved probe is expected to substantially reduce the intra-individual variation in mitoPO_2_ readings.

In this first set of human mitoVO_2_ measurements, under stop flow conditions, we chose to calculate the oxygen consumption by a simple fit of the ΔPO_2_/Δt curve. This resulted in a mitoVO_2_ of around 6 mmHg s^-1^_._ The measured values for mitoVO_2_ in human sternal skin are comparable to the results reported for the abdominal skin of rats [23, 26], i.e. 5.8 ± 2.3 mmHg s^-1^ and 5.0 ± 0.3 mmHg s^-1^ respectively_._

The mitoVO_2_ measurements were performed with a heated measurement probe at 34°C and at 40°C. Although increased oxygen consumption was expected at a higher skin temperature, no significant difference was observed in these experiments.

Fig 4 demonstrated a correlation between mitoVO_2_ and initial mitoPO_2._ This finding is likely due to the used method for analysis of the oxygen disappearance rate, which assumes the measuring volume to be oxygen-tight. However, oxygen diffusion into the measuring volume during an ODR measurement increases at lower mitoPO_2_ and causes the deflection point and deviation from linearity at low mitoPO_2_. A disadvantage of the used linear fit procedure is that it is dependent on the deflection point. At low initial oxygen levels the deflection point has a greater influence on the fitting procedure and gives rise to the correlation between mitoVO_2_ and initial PO_2_. This limitation might be solved by using a different analysis method for the oxygen disappearance rate, which takes oxygen back diffusion into account [24, 34]. Although this more complex method of analyzing has been evaluated for use in rats [24], it has yet to be validated for use in humans; therefore, for the present study, we chose to use the more straightforward linear fit procedure.

The PpIX-TSLT is a novel noninvasive measurement tool that might be of considerable benefit in emergency, intensive care and peri-operative medicine. For example, mitoPO_2_ measurements could be useful to optimize oxygenation or hemodynamic status, or to guide treatment in critically ill patients [21]. PpIX-TSLT provides a new tool for mitochondrial research but does have some limitations. For example, unlike *ex vivo* respirometry, currently it cannot measure quantitatively the bioenergetic state of mitochondria and examine the different states of mitochondrial respiration. Also, measurements in the skin might not be representative of all other tissues in the body. Furthermore, although mitochondria in some tissues (like cardiac muscle) consume approximately 90% of oxygen [35] it is important to recognize that mitochondria are not the sole consumers of oxygen. In this respect it is important to note that in rat skin we found cyanide to be a very effective blocker of oxygen consumption within the measuring volume [23]. Assessment of mitochondrial respiration in vivo is also possible by dynamic near-infrared spectroscopy. In contrast to PpIX-TSLT, NIRS is measured in muscle and results have been demonstrated to correlate with respirometry in muscle biopsies (9). However, NIRS appears somewhat more cumbersome to use for clinical monitoring because of the need for calibration and the use of blood-pressure cuffs.

This study is the first to demonstrate that measurement of mitochondrial parameters by PpIX-TSLT is feasible in humans. Although the technique still needs further development and optimization, the prototype allows evaluation in humans and the clinical setting. Therefore, we conclude that the method itself is feasible to measure mitoPO_2_ and mitoVO_2_ in humans. A follow-up study comparing PpIX-TSLT to other mitochondrial function measurements remains to be performed, but use of PpIX-TSLT in pre-clinical studies showed promising results in the fields of sepsis and transfusion medicine [26, 36] We expect that clinical implementation of the PpIX-TSLT will make a valuable contribution to our knowledge on mitochondrial function and oxygen metabolism under healthy and pathophysiological circumstances. For example, this technique could be a useful tool to evaluate topical skin treatment, for guidance of systemic mitochondrial therapy, and to monitor mitochondrial function in critically ill patients.

## Supporting Information

S1 Appendix Data file of all individual animal experiments. (XLSX)
