# Correction: Cutaneous Respirometry as Novel Technique to Monitor Mitochondrial Function: A Feasibility Study in Healthy Volunteers

**DOI:** 10.1371/journal.pone.0163399

**Published:** 2016-09-15

**Authors:** Floor Anneleen Harms, Robert Jan Stolker, Egbert Gezinus Mik

The first and third authors’ names are spelled incorrectly. The correct first author’s name is: Floor Anneleen Harms. The correct third author’s name is: Egbert Gezinus Mik. The correct citation is: Harms FA, Stolker RJ, Mik EG (2016) Cutaneous Respirometry as Novel Technique to Monitor Mitochondrial Function: A Feasibility Study in Healthy Volunteers. PLoS ONE 11(7): e0159544. doi:10.1371/journal.pone.0159544
